# Draft Genome Sequence of *Alicycliphilus* sp. B1, an *N*-Acylhomoserine Lactone-Producing Bacterium, Isolated from Activated Sludge

**DOI:** 10.1128/genomeA.00424-15

**Published:** 2015-05-14

**Authors:** Noriya Okutsu, Tomohiro Morohoshi, Tsukasa Ikeda

**Affiliations:** a Department of Material and Environmental Chemistry, Graduate School of Engineering, Utsunomiya University, Utsunomiya, Tochigi, Japan; b JST, CREST, Kawaguchi, Japan

## Abstract

We report here the draft genome sequence of *Alicycliphilus* sp. B1, isolated from activated sludge in a wastewater treatment plant of an electronic component factory as an *N*-acylhomoserine lactone-producing strain. The draft genome is 7,465,959 bp in length, with 59 large contigs. About 7,391 protein-coding genes, 82 tRNAs, and 13 rRNAs are predicted from this assembly.

## GENOME ANNOUNCEMENT

The genus *Alicycliphilus* is a member of the family *Comamonadaceae* in the β-subclass of the *Proteobacteria* and comprises a heterogeneous group of motile, oxidase-positive, Gram-negative bacteria (1). *Alicycliphilus denitrificans* strain K601, previously identified as a *Pseudomonas* species, was isolated from a municipal sewage treatment plant with cyclohexanol as the sole carbon source and nitrate as the electron acceptor (2). *A. denitrificans* strain BC was isolated from benzene-degrading chlorate-reducing enrichment (3).

In many Gram-negative bacteria, *N*-acylhomoserine lactones (AHLs) have been identified as signal compounds involved in quorum sensing (4). Many Gram-negative bacteria isolated from activated sludge showed AHL-producing activity (5, 6). In addition, it was reported that AHL-mediated quorum sensing affects the wastewater treatment process that uses activated sludge (5). We isolated *Alicycliphilus* sp. strain B1 as an AHL-producing strain from activated sludge in the wastewater treatment plant of an electronic component factory. However, there have been no reports about the ability of AHL production for the genus *Alicycliphilus*. In a previous study, the complete genome sequences of *A. denitrificans* BC and K601 have been reported (7) and deposited in the DDBJ/EMBL/GenBank database (accession numbers CP002449 to CP002451 and CP002657 to CP002658, respectively). In this study, we determined the draft genome sequence of *Alicycliphilus* sp. strain B1.

Sequencing was performed on a Pacific Biosciences (PacBio) RS instrument (Pacific Biosciences, Menlo Park, CA, USA) using libraries prepared with the SMRTbell template prep kit version 1.0 (Pacific Biosciences) by TaKaRa Bio (Mie, Japan). We produced 209,929 reads with an average read length of 6,414 bases. The total number of sequenced bases is 1,346,455,891, representing a sequencing depth of 25×. The sequencing reads were assembled using the PacBio SMRT Analysis software version 2.2.0 (8). These reads were assembled into 59 large contigs. Prediction of putative coding sequences and gene annotation were done using the Microbial Genome Annotation Pipeline (http://www.migap.org/index.php/en). Briefly, protein-coding sequences (CDSs) were predicted by the combined use of MetaGeneAnnotator (9), RNAmmer (10), tRNAScan (11), and BLAST (12).

The draft genome of the *Alicycliphilus* sp. B1 contains 7,465,959 bp with an average G+C content of 67.32%. The genome contains 7,391 protein-coding genes, 13 rRNA genes, and 82 tRNA genes. We searched for homologues of the reported AHL synthase gene in the complete genome sequence of B1. One predicted coding sequence (ALISP_0667), which encoded 200 amino acids, showed 76% identity with DelCs14_1734 from *Delftia* sp. Cs1-4 (UniPlot accession no. F6AQ77). Further studies on AHL-mediated quorum sensing might contribute to elucidate the mechanisms of gene expression underlying the water treatment of *Alicycliphilus* sp. B1.

### Nucleotide sequence accession number. 

The whole-genome shotgun project of *Alicycliphilus* sp. B1 has been deposited at DDBJ/EMBL/GenBank under the accession number BBSJ00000000. The first version described in this paper has the accession number BBSJ01000000 and consists of sequences BBSJ01000001 to BBSJ01000059.
